# *Escherichia coli* ST302: Genomic Analysis of Virulence Potential and Antimicrobial Resistance Mediated by Mobile Genetic Elements

**DOI:** 10.3389/fmicb.2019.03098

**Published:** 2020-01-21

**Authors:** Veronica M. Jarocki, Cameron J. Reid, Toni A. Chapman, Steven P. Djordjevic

**Affiliations:** ^1^ithree institute, University of Technology Sydney, Sydney, NSW, Australia; ^2^Australian Centre for Genomic Epidemiological Microbiology, University of Technology Sydney, Sydney, NSW, Australia; ^3^NSW Department of Primary Industries, Elizabeth MacArthur Agricultural Institute, Menangle, NSW, Australia

**Keywords:** aEPEC, ST302, mobile genetic elements, IncHI2 plasmid, IncFIB, ETT2, *Escherichia coli*

## Abstract

aEPEC are associated with persistent diarrhea, and diarrheal outbreaks in both humans and animals worldwide. They are differentiated from typical EPEC by the lack of bundle-forming pili, and from EHEC by the lack of phage-mediated *stx* toxins. However, phylogenetic analyses often associate aEPEC with EHEC, promoting the hypothesis that aEPEC are the progenitors of EHEC, which is supported by aEPEC conversion to EHEC by *stx*-carrying phages. While aEPEC can cause disease outright, the potential to acquire *stx*, one of the most potent bacterial toxins known, merits close monitoring. *Escherichia coli* ST302 (O108:H9, O182:H9, O45:H9) are aEPEC that have been isolated from diarrheic human, pig and rabbit hosts, as well as in healthy pigs, however, no study to date has focused on *E. coli* ST302 strains. Through WGS and hybrid assembly we present the first closed chromosome, and two circularized plasmids of an ST302 strain - F2_18C, isolated from a healthy pig in Australia. A phylogenetic analysis placed *E. coli* ST302 strains in proximity to EHEC ST32 (O145:H28) strains. Public databases were interrogated for WGSs of *E. coli* ST302 strains and short-read gene screens were used to compare their virulence-associated gene (VAG) and antimicrobial resistance gene (ARG) cargo. *E. coli* ST302 strains carry diverse VAGs, including those that typically associated with extraintestinal pathogenic *E. coli* (ExPEC). Plasmid comparisons showed that pF2_18C_FIB shared homology with EHEC virulence plasmids such as pO103 while pF2_18C_HI2 is a large multidrug resistance IncHI2:ST3 plasmid. A comparison of 33 HI2:ST3 plasmids demonstrated that those of Australian origin have not acquired resistances to extended-spectrum beta-lactams, colistin, fosfomycin or rifampicin, unlike those originating from Asia. F2_18C was shown to carry two additional pathogenicity islands – ETT2, and the STEC-associated PAI_*CL*__3_, plasmid-associated heavy metal resistance genes, as well as several unoccupied *stx*-phage attachment sites. This study sheds light on the virulence and AMR potential of *E. coli* ST302 strains and informs AMR genomic surveillance.

## Introduction

*Escherichia coli* is a versatile Gram-negative bacterium whose genome is shaped by lateral gene transfer. The plasticity of *E. coli* genomes allows for some strains to exist as gastrointestinal tract commensals, while the acquisition of different combinations of virulence-associated genes (VAGs) has generated clades that cause a diverse range of intestinal and extraintestinal illnesses (Tenaillon et al., 2010). Pathogenic *E. coli* that induce intestinal disease are known as diarrheagenic *E. coli* (DEC) and are classified into pathotypes according to specific virulence factors and ensuing clinical manifestations (Gomes et al., 2016).

Enterohemorrhagic *E. coli* (EHEC) and enteropathogenic *E. coli* (EPEC) are DEC pathotypes that share a common mechanism of pathogenesis defined in part by the carriage of a chromosomally located pathogenicity island (PAI) termed the locus of enterocyte effacement (LEE). The LEE enables EPEC and EHEC to adhere intimately to intestinal epithelial cells and cause attaching and effacing (A/E) lesions leading to diarrheal illness (Kaper et al., 2004). The genes located within the LEE are sufficient for A/E lesion formation, as transferring LEE to commensal *E. coli* confers A/E lesion activity (McDaniel and Kaper, 1997). In addition to the LEE, EHEC and EPEC possess various non-LEE (*nle*)-encoded effector genes. *nle* genes are frequently carried on prophage elements and contribute to virulence by interfering with host signaling pathways, apoptosis and phagocytosis (Wong et al., 2011) as well as disrupting host cell cytoskeleton and tight junctions (Gomes et al., 2016). However, EHEC differ from EPEC in that they possess phage-associated Shiga toxins (*stx*), which contribute to the hemorrhagic colitis and hemolytic uremic syndrome (HUS) associated with EHEC infections (Mayer et al., 2012). EHEC also fall under the category of Shiga toxin-producing *E. coli* (STEC); however, STEC encompass both LEE-positive (typical EHEC) and LEE-negative (atypical EHEC) *E. coli.*

EPEC are further classified into typical EPEC (tEPEC) and atypical EPEC (aEPEC) by the presence, in the former, of the EPEC adherence factor plasmid (pEAF) encoding bundle-forming pili (Gomes et al., 2016). tEPEC and aEPEC also differ in terms of serogroups, reservoirs and virulence factors. tEPEC generally belong to the twelve classical EPEC serogroups recognized by the World Health Organization (O26, O55, O111, O114, O119, O126, O127, O128, O142, O158) (World Health Organization [WHO], 1987). Humans are their principal reservoir (Trabulsi et al., 2002), and their virulence factors are mainly encoded by LEE and pEAF (Abreu et al., 2013). Conversely, aEPEC strains belong to more than 4200 different serotypes (Gomes et al., 2016), and include tEPEC and EHEC that have lost pEAF and *stx* genes, respectively (Afset et al., 2008; Bielaszewska et al., 2008). aEPEC infect both human and animal hosts (Hu and Torres, 2015) and are also more heterogeneous than tEPEC in their virulence factors; frequently carrying genes associated with other DEC pathotypes, including enterohemolysin (*ehxA*) and heat-stable enterotoxin (*astA*) (Bando et al., 2009).

aEPEC strains have been associated with persistent diarrhea in both humans (Hu and Torres, 2015) and animals (Watson et al., 2017; Arais et al., 2018) and in diarrheal outbreaks in Brazil, China, Finland, Japan, and the United States (Hu and Torres, 2015; Gomes et al., 2016). While recent epidemiological studies have indicated that aEPEC is more prevalent than tEPEC in both industrialized and developing countries (Hu and Torres, 2015), aEPEC pathogenicity remains controversial due to similar isolation rates from both diarrheic and non-diarrheic hosts (Gomes et al., 2016). Establishing a baseline for which virulence factors may or may not contribute to disease can help elucidate the mechanisms behind aEPEC pathogenicity.

*Escherichia coli* is not only an agent of disease, but also a driving force behind antimicrobial resistance (AMR). In fact, *E. coli* is one of the most significant global concerns in human and animal health sectors, the food industry and in the environment (Paitan, 2018). AMR surveillance programs have indicated that resistance to all the major classes of antibiotics now circulate among *E. coli* strains (Pitout, 2012), including extended-spectrum β-lactams (ESBL), carbapenems, and more recently, plasmid-mediated colistin resistance (*mcr-1*), particularly in food animals from China and Vietnam (Liu et al., 2016; Huang et al., 2017; Caruso, 2018). The ubiquitous nature of *E. coli* means it constitutes a shared reservoir for AMR across a One Health framework, and concerns have been raised about the possible transmission of AMR *E. coli* between animals and humans through direct contact or via the food chain (Poirel et al., 2018). As inter- and intraspecies horizontal gene transfer (HGT) and mobile genetic elements (MGE) are considered the prevailing mechanisms that drive AMR (Tzouvelekis et al., 2012; Partridge et al., 2018), close genomic surveillance of AMR cargo within *E. coli* populations is warranted.

aEPEC from sequence type (ST) 302 (serotype O108:H9) have been isolated from both healthy and diseased hosts, and the environment. They have been isolated from healthy pigs (Fröhlicher et al., 2008; Malik et al., 2017; Reid et al., 2017), water tanks in a poultry slaughterhouse (Alonso et al., 2014) and pork products made for human consumption (Lugsomya et al., 2018), and have been associated with diarrheic rabbits (Zhao X. et al., 2018), pigs (Kleta et al., 2014), and humans (Foster et al., 2015). Yet to date, no genomic comparisons of *E. coli* ST302 strains, nor a complete ST302 genome, has been published. Here we sequenced and annotated the complete genome of *E. coli* aEPEC strain F2_18C (ST302, O108:H9), isolated from a healthy pig not previously treated with antibiotics on an Australian farm. We performed comparative analyses with other *E. coli* ST302 strains, screening for virulence and AMR cargo, and compared F2_18C’s three plasmids – an HI2:ST3 plasmid with homology to colistin-resistant plasmids, an FIB plasmid with homology to the EHEC virulence plasmid, and a colRNAI plasmid carrying colicin-10 – to other plasmids with high sequence identity.

## Materials and Methods

### Ethics Statement

NSW Department of Primary Industries did not require the study to be reviewed or approved by an ethics committee as *E. coli* strain F2_18C was isolated from a veterinary sample provided by the farm veterinarian as part of routine diagnostics.

### Isolation and Storage of *E. coli* Strain F2_18C

Strain F2_18C was isolated from a commercial-raised, healthy piglet (weaner), not previously treated with antibiotics, on an Australian farm as previously described (Reid et al., 2017). The strain was cultured in LB medium, frozen as a glycerol stock (500 μl M9 salts solution, 500 μl 50% v/v glycerol) and stored at −80°C. The strain was again cultured in LB medium before total cellular DNA isolation for sequencing.

### DNA Extraction, Whole-Genome Sequencing and Assembly

For short-read sequencing, DNA was extracted using the ISOLATE II genomic DNA kit (Bioline) and stored at −20°C. Short-read sequencing was performed using an Illumina HiSeq 2500 v4 sequencer in rapid PE150 mode as previously described (Reid et al., 2017). For long-read sequencing, DNA was extracted from mid-log phase sub-cultures of strains grown overnight in LB broth using a gentle phenol-chloroform extraction protocol. Long-read sequencing was performed by the Ramaciotti Centre for Genomics using a Pacific Bioscience (PacBio) RSII sequencer with P6-C4 chemistry. Sequence read quality was assessed using FastQC version 0.11^1^. A hybrid assembly, using both Illumina and PacBio reads, was produced using Unicycler (Wick et al., 2017).

### Genome Annotation

Automated annotations were generated by RASTtk (Brettin et al., 2015) and managed using SnapGene v4.1.9. Manual annotations were added using publicly available databases and tools: insertion sequences (IS) were annotated manually using ISfinder (Siguier et al., 2006)^2^; genomic islands (GIs) were identified using IslandViewer 4 (Bertelli et al., 2017)^3^; functional assignments of ORFs were designated using the Comprehensive Genome Analysis service available through Pathosystems Resource Integration Center (PATRIC v3.5.41) (Wattam et al., 2017)^4^ and; phage elements were identified using Phage Search Tool Enhanced Release (PHASTER) (Arndt et al., 2016)^5^. Sequences for the F2_18C chromosome, pF2_18C_HI2, pF2_18C_FIB, pF2_18C_Col have been deposited to NCBI under the accession numbers CP043542, CP043545, CP043544, and CP043543, respectively, and annotated using the NCBI automated pipeline. Authors’ annotations (GenBank files) can be viewed in Supplementary Data S1.

### Phylogenetic Analysis

Maximum-likelihood phylogenetic distances between genomes were analyzed using Phylosift (Darling et al., 2014), generated using FastTree2 (Price et al., 2010), and visualized using both FigTree v1.4.2^6^ and the Interactive Tree of Life (iTOL v4) (Letunic and Bork, 2019)^7^. SNP-based phylogenetic analysis was performed using Snplord, an automated pipeline that utilizes snippy^8^, Gubbins^9^, SNP-sites^10^, and FastTree 2. Snplord is available at https://github.com/CJREID/snplord. The ST302 pangenome was calculated by Roary v3.11.2 (Page et al., 2015).

### Genotyping, Serotyping, and Phylogenetic Classification

F2_18Cs ST, serogroup and phylogroup were determined *in silico* using MLST 2.0 (Larsen et al., 2012)^11^, SerotypeFinder 2.0 (Joensen et al., 2015)^12^ and ClermontTyping (Clermont et al., 2013)^13^, respectively. To compare VAG, AMR gene and plasmid replicon carriage between F2_18C and other *E. coli* ST302 strains, short-reads of publicly available *E. coli* ST302 strains were first downloaded from Enterobase (Alikhan et al., 2018; downloaded 01/09/18)^14^ and then screened using ARIBA read-mapping tool (Hunt et al., 2017), using the following reference databases: ResFinder (Zankari et al., 2012)^15^, CARD (Jia et al., 2017)^16^, VirulenceFinder (Joensen et al., 2014)^17^, VFDB (Liu et al., 2019)^18^, and PlasmidFinder (Carattoli et al., 2014)^19^. ARIBA data was processed using a bespoke script accessible at https://github.com/maxlcummins/ARIBAlord.

### Plasmid and Pathogenicity Island Comparisons

Plasmids with high sequence identity (>98%) were found through the PLSDB v2019_06_03 (Galata et al., 2019)^20^ and comparative analyses were achieved using BRIG (Alikhan et al., 2011)^21^ and Mauve (Darling, 2004)^22^. Mauve and Easyfig (Sullivan et al., 2011) were used to compare PAI_*CL*__3_ and ETT2 pathogenicity islands.

### Phenotypic Resistance Testing

F2_18C phenotypic resistance was tested using the calibrated dichotomous susceptibility (CDS) test (Bell et al., 2016) against the following antibiotics: ampicillin (25 μg), azithromycin (15 μg), cefoxitin (30 μg), ciprofloxacin (2.5 μg), chloramphenicol (30 μg), gentamicin (10 μg), imipenem (10 μg), kanamycin (50 μg), nalidixic acid (30 μg), neomycin (30 μg), streptomycin (25 μg), sulphafurazole (300 μg), tetracycline (10 μg), and trimethoprim (5 μg).

## Results

### General Genomic Features of *E. coli* Strain F2_18C

**FIGURE 1:**
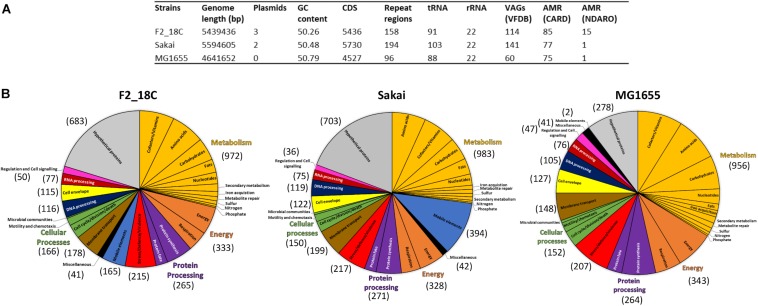
General features of strain F2_18C compared to *E. coli* strains Sakai and MG1655. **(A)** Table listing general genomic features. **(B)** Subsystem class gene distributions. Number in brackets represent number of genes assigned to each functional category.

*Escherichia coli* strain F2_18C has a circular genome of 5,439,436 bp consisting of 5436 CDS, 158 repeat regions, 91 tRNA and 22 rRNA. Virulence Factor Database (VFDB) identified 114 VAGs, and the Comprehensive Antibiotic Resistance Database (CARD) identified 85 antimicrobial resistance (AMR) genes. The National Database of Antibiotic Resistance Organisms (NDARO) database identified 15 AMR genes (Figure 1A). In terms of functional properties, strain F2_18C has 4753 genes encoding proteins with functional assignments and 683 hypothetical proteins. The three categories in which the most genes are attributed to are metabolism (*n* = 972), energy (*n* = 333), and protein processing (*n* = 265) (Figure 1B).

When compared to a highly pathogenic *E. coli* strain (O157:H7 strain Sakai), and a non-pathogenic *E. coli* strain (laboratory strain MG1655), strain F2_18C had fewer VAGs than Sakai (*n* = 141) but more than MG1655 (*n* = 60). However, F2_18C had more AMR genes than both Sakai (77 CARD/1 NDARO) and MG1655 (75 CARD/1 NDARO). An interesting observation regarding differences in functional properties between the three strains was the number of genes associated with mobile elements (prophages, transposable elements and plasmids) – 165 for F2_18C, 394 for Sakai, and 2 for MG1655. A complete list of F2_18C gene functional assignments (GO assignments, subsystems, and PL/PGfam) and specialty genes (VAGs, transporters, drug targets, and AMR genes) can be viewed in Supplementary Table S1.

### Phylogenetic Analysis

**FIGURE 2:**
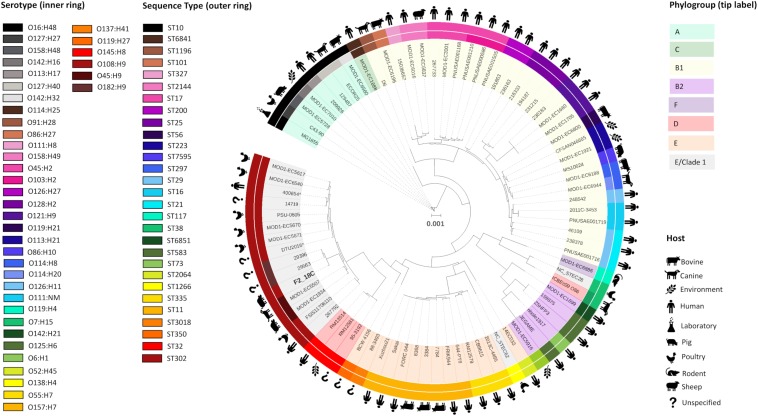
Maximum-likelihood phylogenetic tree showing genetic relatedness of *E. coli* strain F2_18C. Phylosift, FastTree2, FigTree and iTOL were used to generate and visualize a maximum-likelihood phylogenetic tree, rooted on *E. coli* laboratory strain MG1655. A total of 91 *E. coli* strains were used, including all publicly available *E. coli* ST302 strains. The colored bands that form the outer ring relate to each strains ST and the second ring refers to serotype. Icons around the circumference refer to the host or environment from which the strains were isolated. Shading over tip labels indicates phylogroups. F2_18C is shown in bold. ^∗^Indicates an abbreviated strain name: 400654^∗^ = Escherichia_coli_400654-sc-2012-01-13T20:11:31Z-1332520; DTU2016^∗^ = DTU2016_ 526_ PRJ1050_ Eschericha_coli_Isol133.

Porcine *E. coli* strain F2_18C was determined to be ST ST302, serotype O108:H9 and phylogroup E or clade I by Achtman 7 MLST, *in silico* serotype prediction and *in silico* Clermont typing, respectively. To determine the genetic relatedness of F2_18C to other *E. coli* ST302 strains and to other *E. coli* strains in general, all publicly available ST302 genomes (*n* = 14), and 67 *E. coli* genomes of varying STs, serotypes, and phylogroups were used to construct a phylogenetic tree (Figure 2; details of analyzed genomes found in Supplementary Table S2). Where known, the ST302 genomes analyzed in this study were from a range of hosts, including pigs, poultry, companion animals and humans, and from broad geographical locations, including Australia, Canada, Kenya, Slovakia, the United Kingdom, and the United States. Tree topology demonstrated consonance between STs, serotypes and phylogroups. The analysis showed that *E. coli* ST302 strains are most closely related to a branch of ST32s (serotype O145:H28, phylogroup D) and that they all belong to the E or cryptic clade 1 phylogroup and carry the H9 antigen. However, three ST302 serogroups were identified – O182, O45, and O108 – the latter being the most common (*n* = 11; 73%). Additionally, F2_18C and two O182:H9 strains (29396 and 29563) formed an offshoot branch from the other ST302 strains. An ST302 SNP analysis demonstrated that F2_18C was separated from its closest relative (29396) by 684 SNPs, and its most distant relative (FSIS11706110) by 2139 SNPs. SNP analysis details, including SNP sites and distribution, can be viewed in Supplementary Data S2. The ST302 pangenome comprised a total of 10262 genes, with 3558 genes forming the core genome (genes present in 100% of ST302 strains), and 6704 genes forming the accessory genome (shell genome [15–95% ST302 strains] = 2587 genes; cloud genome [<15% ST302 strains] = 4117 genes). Pangenome details, including gene absence/presence table and Roary-generated graphs, can be viewed in Supplementary Table S3.

### ST302 Gene Screening

**FIGURE 3:**
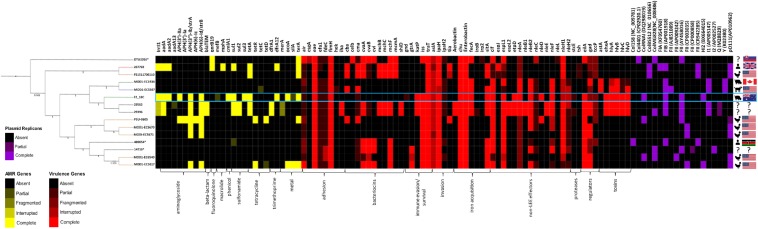
Heatmap and SNP tree of ST302 virulence factors, antimicrobial resistance and plasmid replicons. Intact genes are represented in solid colors, while partial, fragmented or interrupted genes are less opaque. Partial, fragmented and interrupted genes encompass all non-intact genes. ARGs and heavy metal resistance genes are shown in yellow, VAGs in red, and plasmid replicons in purple. Host and country of origin depicted on the right. F2_18C boxed in blue. Tree branch colors are representative of host – red = human; yellow = poultry; green = canine; blue = porcine; black = unspecified. ^∗^Indicates an abbreviated strain name: 400654^∗^ = Escherichia_coli_400654-sc-2012-01-13T20:11:31Z-1332520; DTU2016^∗^ = DTU2016_526_ PRJ1050_ Eschericha_coli_Isol133. The phylogenetic tree was produced by SNP-based phylogenetic analysis.

All *E. coli* ST302 strains, including strain F2_18C, were screened for acquired antimicrobial resistance genes (ARGs), heavy metal resistance genes, VAGs and plasmid replicons using Ariba. The text below refers to genes presented in Figure 3, which is a selection of significant VAG and ARG, the full list of screened genes can be viewed in Supplementary Table S4.

#### Acquired ARGs and Heavy Metal Resistance Genes

In *E. coli* ST302 strains, genes conferring resistance to aminoglycosides, beta-lactams, fluoroquinolones, macrolides, phenicols, sulfonamides, tetracycline and trimethoprim were detected (Figure 3). No genes conferring resistance to extended-spectrum beta-lactams and carbapenems were detected in any *E. coli* ST302 strains. Strain F2_18C had the greatest number of ARGs (11 versus an average of 3), was 1 of 4 strains carrying resistance genes to 3 or more classes of antibiotics, and was 1 of 2 strains with an intact *intI1*, the other belonging to strain 267792, isolated from a human in the United Kingdom. The most common acquired ARGs across *E. coli* ST302 strains were streptomycin resistance genes *strA* and *strB* (*n* = 7; 47%), tetracycline resistance gene *tetA* (*n* = 5; 33%), aminoglycoside resistance gene *aadA1* (*n* = 4; 27%), and sulfonamide resistance gene *sul1* (*n* = 4; 27%). In terms of heavy metal resistance genes, *E. coli* ST302 strains carry intact copper resistance gene *pcoA* (*n* = 2; 13%), silver resistance gene *silA* (*n* = 1; 7%), and tellurite resistance gene *terA* (*n* = 6; 40%).

Strain F2_18C phenotypic resistances and ARGs were congruent. F2_18C was resistant to sulphafurazole (*sul1, sul3*), tetracycline (*tetA*), streptomycin (*strA, strB*), chloramphenicol (*cmlA*), kanamycin and neomycin [*aph(3’)-IIa*], and trimethoprim (*dfrA12*). F2_18C also possesses two additional aminoglycoside resistance genes *aadA1* and *aadA2*.

#### Virulence-Associated Genes

All *E. coli* ST302 strains possess the LEE pathogenicity island adhesin gene *eae* (β variant in F2_18C) but lack Shiga toxin genes (*stx*), and bundle-forming pili (BFP) genes, broadly classifying them as aEPEC. Strain F2_18C had the most VAGs of all the O108:H2 serotypes (*n* = 23, versus an average of 14) (Figure 3). However, the two O182:H9 strains, 29563 and 29396, which together with F2_18C, form an offshoot from other *E. coli* ST302 strains (Figure 1), had the highest number of VAGs overall (*n* = 33 each). The additional VAGs seen in these two strains, but not in F2_18C include adhesin *iha*, bacteriocins *cvaA, mchB, mchC, mchF, mcmA, shiD*, aerobactin iron acquisition genes (*iucABCD*, *iutA*), and non-LEE effectors *espL2, nleB1* and *nleE*. Strains F2_18C, 29563 and 29396 were the only *E. coli* ST302 strains to possess EHEC virulence plasmid associated genes *ehxA, etpD* and *katP.*

Regarding adherence, in addition to aforementioned *eae, iha* and *fimH*, two strains, DTU2016 and MODI-EC5617, possess an intact EAEC adhesin encoding gene *air*, and all other strains have at least partial hits to this gene. Most strains also have at least partial *efa1* (EHEC factor for adherence; *n* = 14; 93%) and *fdeC* (factor adherence *E. coli*; *n* = 13; 87%) genes. However, they all lack *paa* (porcine attaching-effacing associated protein) and plasmid-associated *toxB* genes. Regarding iron uptake, all *E. coli* ST302 strains possess *E. coli* heme uptake (*chu*) and enterobactin genes (*ent*, *fep*). Only the aforementioned O182:H9 strains had aerobactin genes, however several strains, including F2_18C, also possess *fecA* (*n* = 6; 40%) and *sitA* (*n* = 10; 67%). *E. coli* ST302 strains carry an array of non-LEE effector genes, the most common being *cif* (cycle inhibiting factor; *n* = 12; 80%), *espL1* (*n* = 12; 80%), and *nleH1* (*n* = 10; 67%), and the least common being *nleD* (*n* = 2; 13%), which F2_18C possesses, and *nleE* (*n* = 2; 13%). In terms of immune evasion and survival, *E. coli* ST302 strains possess *iss* (increases serum survival; *n* = 11; 73%), *traT* (*n* = 15, 100%), and *gtrA* (*n* = 2; 13%). For invasion, ST302 have *ipaH* (*n* = 14; 93%), *tia* (*n* = 2; 13%), and all have at least partial *aslA.* Several *E. coli* ST302 strains have hemolysin toxin genes, and two strains (MOD1-EC1934 and MOD1-EC5507) also possess *astA* (heat-stable enterotoxin 1/EAST-1). As ARIBA gene screening utilizes Illumina short reads only, the F2_18C chromosome was interrogated manually, using BLASTp and VFDB for reference sequeneces, for gene products flagged as not intact (only VAGs absent in laboratory *E. coli* strain MG1655 were searched). The amino acid (a.a) sequence identity of these ORFs compared to pathogenic *E. coli* strains are shown in Table 1, and EPEC and/or EHEC VAGs absent in F2_18C are shown in Table 2.

**TABLE 1:** Interrogated F2_18C chromosomal VAGs.

**Function**	**Gene**	**% a. a sequence identity**	**Compared *E. coli* strain**	**VAG Pathotype**	**ARIBA output**	**Comments**
Adhesion	*cgsA-G*	98.55-100	EC958	UPEC	Missing	False-negative
	*eaeH*	61.21	Sakai	Multiple	Missing	Split into 2 ORFS, missing N and C termini, 146 kDa in F2_18C vs. 150 kDa in Sakai
	*aidA-1*	96.63	Sakai	Multiple	Missing	False-negative
	*upaG*	86.76	Sakai	Multiple	Missing	False-negative
	*air*	55.04	042	UPEC, EAEC, NMEC	Partial	Missing 117 kDa fragment
Non-LEE encoded effectors	*tccP*	71.51	Sakai	EHEC, EPEC	Fragmented	Missing middle section, 33 kDa in F2_18C vs. 37 kDa in Sakai
	*efa1*	5.616	11128	EHEC, EPEC	Partial	C-terminus only (100% identity), 21 kDa in F2_18C vs. 366 kDa in 11128
	*espY2*	51.08	Sakai	EHEC, EPEC	Missing	Additional C-terminus, 31 kDa in F2_18C vs. 21 kDa in Sakai
	*espY3*	87.00	Sakai	EHEC, EPEC	Interrupted	Split into 2 ORFs
	*nleC*	24.78	Sakai	EHEC, EPEC	Partial	N-terminus only, 11 kDa in F2_18C vs. 37 kDa in Sakai
	*nleG2-2*	77.49	Sakai	EHEC, EPEC	Missing	
	*nleH1-1*	97.95	Sakai	EHEC, EPEC	Interrupted	False-negative
	*nleH1-2*	49.51	Sakai	EHEC, EPEC	Interrupted	Missing N and C termini, 21 kDa in F2_18C vs. 34 kDa in Sakai
Invasion	*ibeB*	99.13	Sakai	Multiple	Missing	False-negative
	*ibeC*	72.96	11368	Multiple	Missing	False-negative
Toxin	*clyA/hlyE*	98.69	Sakai	Multiple	Missing	False-negative

**TABLE 2:** EPEC/EHEC VAGs absent in F2_18C.

**Function**	**VAG present in some EPEC and/or EHEC strains**
Adhesion	*paa, lpfA, toxB, perA*
Non-LEE encoded effectors	*espJ, espK, espL2, espM1-2, espN, espO, espW, espX6-X7, espY1, espY5, nleB1, nleB2-2, nleE, nleG, nleG2-G8*
Serine Protease Autotransporters of Enterobacteriaceae (SPATE)	*ehaC, espP, pet, sepA, tsh*
Secretion	ACE T6SS (*aec* genes)
Toxins	*astA, sen, stx*

#### Plasmid Replicons

All *E. coli* ST302 strains were identified as possessing at least two plasmid replicons (Figure 3). Various Col plasmids were identified in 9 strains (60%), FIA in 1 strain, FIB in 7 strains (47%), various FII in 14 strains (93%), HI2 in 2 strains (13%), I1 in 4 (27%), I2 in a single strain, Y in 2 strains (13%) and pO111 in 6 (40%). FII, FIB, HI2 and ColRNAI replicons were identified in F2_18C.

### F2_18C Mobile Genetic Elements

#### F2_18C Chromosomal MGEs

The F2_18C chromosome has 52 intact ISs with IS*1203* (and IS*1203*-like) occurring the most frequently (*n* = 23), followed by a novel IS with some homology to IS*Kpn28* (*n* = 7, 85% sequence identity to IS*Kpn28*), and IS*4* (*n* = 6). Several IS*1203*s disrupt ORFs, including a DNA protecting protein DprA (458183.459396), *N*-acetylgalactosamine-6-phosphate deacetylase (600929.602142), D-glucarate transporter (1032798.1034011), lipoprotein YnfC (2369004.2370217) and invasion plasmid antigen (2788262.2789475).

**FIGURE 4:**
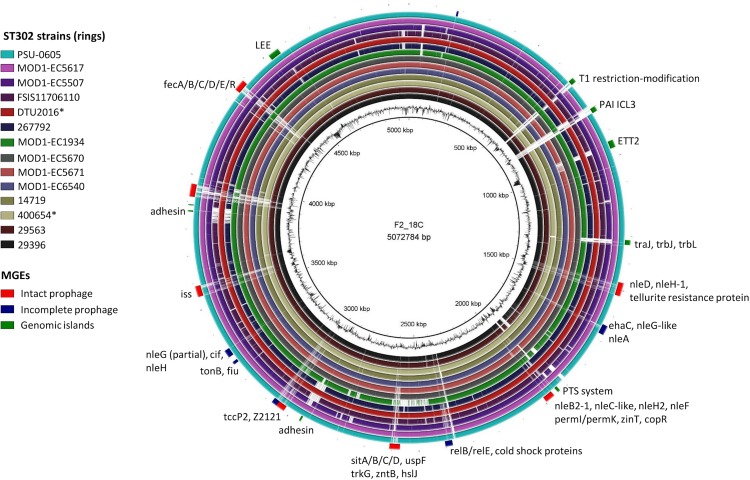
BRIG diagram of MGE distribution in the F2_18C chromosome compared to other ST302 strains. Along the periphery, intact prophages are shown in red, incomplete prophages in navy and GIs in green. Some notable genes present in these regions are labeled accordingly. ^∗^Indicates an abbreviated strain name: 400654^∗^ = Escherichia_coli_400654-sc-2012-01-13T20:11:31Z-1332520; DTU2016^∗^ = DTU2016_526_ PRJ1050_ Escherichia _coli_Isol133.

When comparing F2_18C with other ST302 strains, MGEs contribute significantly to chromosomal variations (Figure 4). PHASTER identified four intact prophages, three questionable, and six incomplete prophages within the F2_18C chromosome and these carry several VAGs such as non-LEE effector genes (*nleB2-1*, *nleC*-like, *nleF*, *nleD*, partial *nleG*, *nleH*, *nleH-1*), Tir-cytoskeleton coupling protein *tccP2*, increased serum survival *iss*, cycle inhibiting factor *cif*, and Z2121 (pathogenicity island OI-57 protein). *nleA nleF* and *nleH1-1* are marker genes for pathogenicity island OI-71, however in F2_18C they are carried by various phage elements (Figure 4). The phage elements also carry several heavy metal-associated genes such as *sitABCD* (iron transport), *fecABCDR* (iron transport), *tonB* and *fiu* (iron transport), *zinT* (zinc binding), *zntB* (cadmium, zinc, and copper resistance), *copR* (copper resistance), and *trkG* (potassium uptake). PHASTER output is provided in Supplementary Data S3.

**FIGURE 5:**
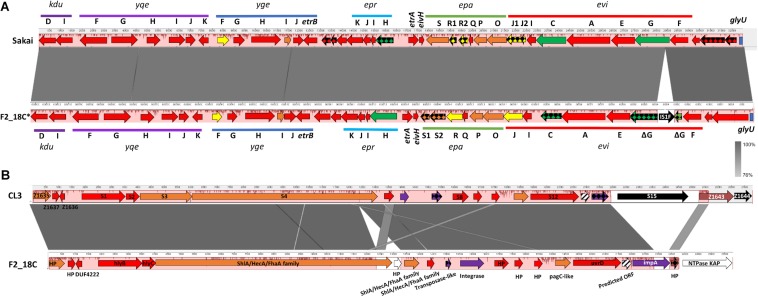
F2_18C pathogenicity island comparisons. Mauve and Easyfig alignments and SnapGene annotations depicting **(A)** the ETT2 regions in *E. coli* strains Sakai and F2_18C (F2_18C sequence is depicted in a reversed orientation) and **(B)** the PAI_*CL*__3_ regions in strains CL3 and F2_18C. The arrow colors represent% amino acid similarity: red = > 95%, orange = > 90%, yellow = > 85%, green = > 60%, purple = > 45%, brown = < 34%, white = ORF not present in CL3, black = ORF not present in F2_18C, checkered = pseudogenes, striped = ORF predicted in F2_18C.

IslandViewer4 was used to identify genomic islands (GIs). The GIs predicted by at least two algorithms are depicted in Figure 4, and a full list can be viewed in Supplementary Table S5. Notably, F2_18C possesses an intact LEE pathogenicity island, with β1-eae and β-tir, and LEE-encoded T3SS effectors. The PAI is attached at tRNA-Phe. F2_18C also possesses a near-intact *E. coli* type 3 secretion system island 2 (ETT2) (Figure 5A). This island in F2_18C has fewer pseudogenes than in *E. coli* strain Sakai (3 vs. 5) however *epaS* is split into two ORFs, EivC is truncated at the C-terminus by 13.7 kDa (120 amino acids), and an IS*1F* insertion which interrupts *eivG*, removing 18 kDa (163 amino acids) off the product’s N-terminus. Furthermore, F2_18C possesses a near-complete PAI_*CL*__3_ pathogenicity island containing adhesin, hemolysin and pagC-like genes. When compared to the original PAI_*CL*__3_ found in *E. coli* O113:H21 (STEC) strain CL3, F2_18C PAI_*CL*__3_ is smaller by 3629 bp (23668 bp vs. 27297 bp). This is largely due to missing the right-side boundary genes S15 and Z1644, and only possessing a fragment of Z1643 (Figure 5B). Other notable differences are as follows: the *Yersinia pestis* ShlA/HecA/FhaA family adhesin (S3/S4 in CL3) appears to be split into 3 ORFs in F2_18C and is larger by 277 amino acids than in strain CL3; the transposase-like factor is truncated at the N-terminus and is smaller by 6.8 kDa (58 amino acids) than the equivalent S6 in CL3.; integrase (S7 in CL3) is extended at the N-terminus making it 18.7 kDa (160 amino acids) larger than S7 and; type VI secretion related protein ImpA does not appear truncated at the N- or C-terminus as it does in CL3, making it 25.7 kDa (227 amino acids) larger than S14. Immediately downstream to the F2_18C PAI_*CL*__3_ right-side boundary is a phage T7 exclusion protein, NTPase KAP (Figure 5B), which may contribute to the aforementioned missing S15 and Z1644 ORFS. The F2_18C PAI_*CL*__3_ isoform is not present in any other ST302 (Figure 4).

As aEPEC can be converted to EHEC by the presence of *stx*-carrying bacteriophages, the F2_18C chromosome was also checked for potential *stx1* and *stx2* integration sites (Bonanno et al., 2015), and was found to have the following unoccupied: *wrbA* (2984895.2985491), *yehV* (1780350.1781081), *yecE* (2076464.2077282), *sbcB* (1921208.1922635), *Z2577* (2354785.2354802) and the *torS/torT* intergenic region (2996339.2996467). Additionally, F2_18C possesses the lambda phage receptor gene LamB (4667691.4669028) required for lambda bacteriophage adsorption (Bertozzi Silva et al., 2016). F2_18C also possesses the *argW* integration site (1521099.152173); however this site is occupied by a non-*stx-*carrying lambdoid prophage.

#### F2_18C AMR Plasmid

**FIGURE 6:**
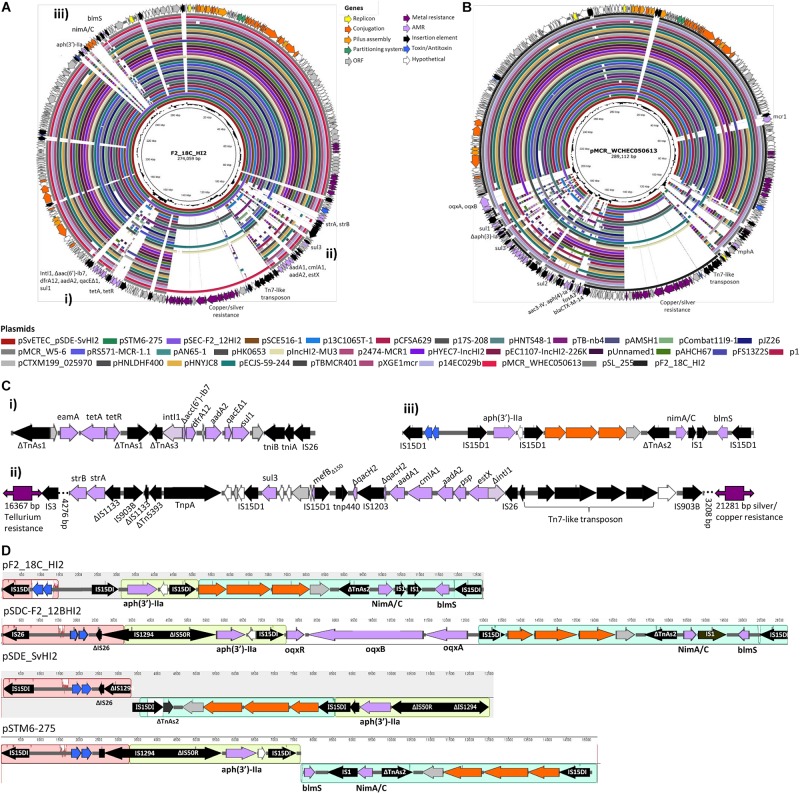
Plasmid maps and BRIG comparison of HI2:ST3 plasmids. **(A)** Plasmid map of pF2_18C_HI2 and alignments of other HI2:ST3 plasmids. **(B)** Plasmid map and alignments of pMCR_WHEC050613 from *E. coli* strain WCHECO50613 isolated from sewerage in China. Plasmid maps show gene annotations and are color coded by function (legend top center). **(C)** Gene structures of F2_18C AMR regions, flagged in **(A)**. **(D)** Mauve alignment of region iii found in other HI2:ST3 isolates originating from Australia.

Unicycler hybrid assemblies resolved plasmid pF2_18C_HI2 as a 274, 059 bp circular sequence with 343 CDSs (163 with functional assignments and 180 hypothetical proteins). It carries 17 IS elements, with IS*26* variant IS*15DI* being the most common (*n* = 8), followed by IS*903B* (*n* = 3). The plasmid was typed using pMLST as incompatibility group HI2, sequence type 3 (HI2:ST3). A full plasmid map is presented in Figure 6A, along with content comparisons to high sequence identity (>98%) matched HI2:ST3 plasmids publicly available (*n* = 33). pF2_18C_HI2 has three regions of AMR carriage. One region comprises a class 1 integron (*intI1*) located within a *Tn172/Tn21* hybrid tetracycline resistance (*tetA*, *tetR*) transposon that has acquired *dfrA12* (trimethoprim resistance) and *aadA2* (aminoglycoside resistance) cassettes with *qacE*Δ*1* (small multidrug resistance efflux transporter), and *sul1* (sulfonamide resistance) genes (Figure 6Ci). A second region contains a truncated *sul3*-associated class 1 integron structure, located near a *Tn7*-like transposon mobilizing copper and silver (*sil*/*pco*) resistance genes at one end and a tellurium resistance genes (*ter*) at the other. The atypical *sul3*-associated integron encodes *estX* (aminoglycoside resistance), *psp* (phosphoserine phosphatase), *aadA2* (aminoglycoside resistance), *cmlA* (phenicol resistance) and *aadA1* (aminoglycoside resistance) cassettes. Further upstream lies a novel truncated version of macrolide resistance gene Δ*mefB*_150_, *sul3* (sulfonamide resistance) and *strA* and *strB* (aminoglycoside resistance) genes (Figure 6Cii). The third region (Figure 6Ciii) is flanked by direct IS*15DI* and contains *aph(3′)-IIa* (neomycin, kanamycin, paromomycin, butirosin, gentamicin B and ribostamycin resistance), bleomycin resistance gene *blemS*, and a NimC/NimA family gene (nitroimidazole reductase) as well as *relE*/*relB* toxin/antitoxin genes and three conjugation proteins, *traO, traF*, and *traG*. This third region appears unique to three other Australian isolates (Figure 6A) and may represent a new IS*15DI*-mediated transposon that has evolved in Australia. However, these regions have undergone some inversions and rearrangements, and acquired probable IS1*5DI*-mediated fluoroquinolone resistance genes *oqxABR* in pSDC-F2_12BHI2 (Figure 6D).

**FIGURE 7:**
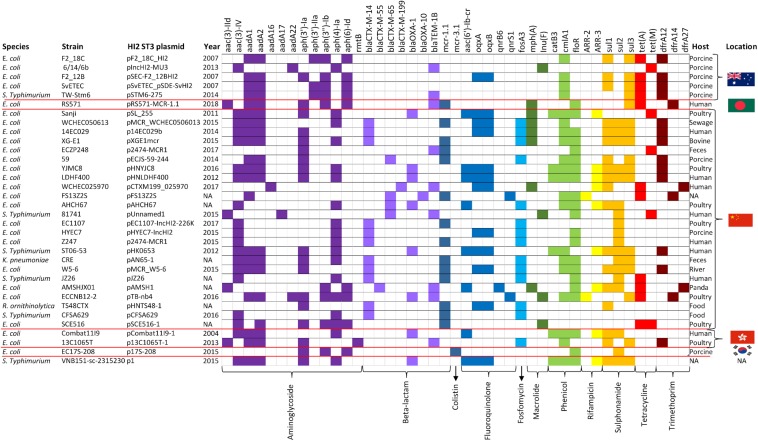
HI2:ST3 AMR gene carriage profiles. AMR genes present in the 33 HI2:ST3 plasmids analyzed in Figure 6. Flags on the right represent isolation location and from top to bottom are Australia, Bangladesh, China, Hong Kong, and South Korea.

The three AMR regions account for most of the structural difference between pF2_18C_HI2 and other HI2:ST3 plasmids (Figure 6A). However, HI2:ST3s carry a smorgasbord of diverse AMR genes (Figures 6B, 7). Currently, no HI2:ST3 plasmid originating from Australia carries any extended-spectrum beta lactams (ESBLs), colistin, fosfomycin or rifampicin resistance genes, unlike HI2:ST3 plasmids originating from Asia (Figure 7).

#### F2_18C Virulence Plasmid

**FIGURE 8:**
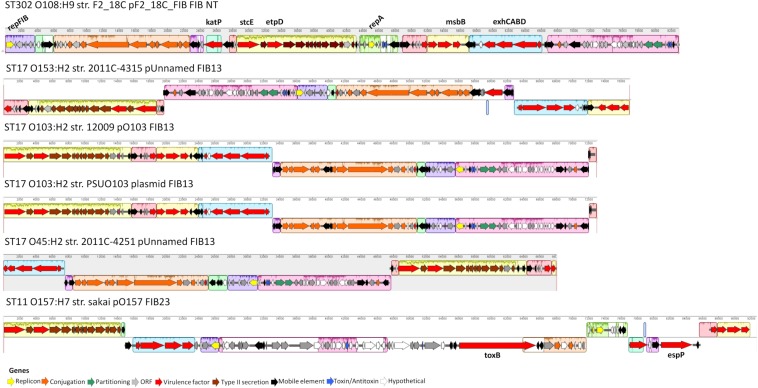
pF2_18C_F plasmid map and FIB13 plasmid alignments. Plasmid maps showing gene annotations for pF2_18C_FIB and four FIB13 plasmids and one FIB23 plasmid over a mauve alignment to demonstrate homologous regions.

Unicycler hybrid assemblies resolved pF2_18C_FIB as a circular sequence of 82, 910 bp containing 100 CDSs (76 with functional assignments, and 24 encoding hypothetical proteins). pF2_18C_FIB contains 6 IS elements with IS*1203* (IS*629* variant) being the most common. This plasmid could not be typed by pMLST, however, it has >99% sequence identity with four FIB13 plasmids, all of which associate with *E. coli* ST17 but of varying serotypes (O45:H2, O153:H2 and two O103:H2). The difficulties in typing may be attributed to an additional RepA replicon in pF2_18C_FIB, which is also seen in pO157 found in O157:H7 strains (Figure 8). In terms of virulence, like the FIB13 plasmids, pF2_18C_FIB carries hemolysin encoding genes (*ehxA*, *hlyB*, *hlyC*, *hlyD*), lipid A modification/synthesis genes (including *msbB*), *stcE* (secreted protease of C1 esterase inhibitor from EHEC), and EHEC associated gene *etpD.* pF2_18C_FIB also carries the catalase gene *katP*, only seen in one other FIB13 plasmid; however, the F2_18C *katP* gene is more homologous to *katP* from pO157 (Figure 8). These plasmids have undergone considerable inversions and rearrangements, which may be the result of the IS elements that flank most regions (Figure 8). pF2_18C_FIB and the FIB13 plasmids share homology to EHEC plasmid pO157 (66% coverage at 90% identity); however, pO157 (from strain Sakai) is 12,688 bp larger than pF2_18C_FIB and contains additional virulence factors including the *toxB* gene and serine protease *etpP* (Figure 8).

#### F2_18C Col Plasmid

**FIGURE 9:**
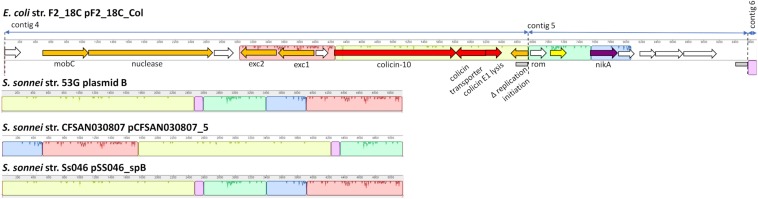
F2_18C contig annotation with mauve alignments to ColRNAI plasmids. Three F2_18C contigs were aligned to ColRNAI_1 plasmids from *S. sonnei* strains (∼98% identity through PLSDB). Homologous regions are shown in color. Two gray boxes within the annotation denote repeat regions.

Three F2_18C contigs did not circularize; however, they have homology to *Shigella sonnei* ColRNAI plasmids (Figure 9). Like the *S. sonnei* plasmids, F2_18C contig 4 carries colicin-10, the colicin transporter, colicin lysis protein, a partial replication initiation protein and two plasmid exclusion proteins, but additional carries a mobC family plasmid mobilization relaxome protein (100% amino acid sequence identity to *E. coli* mobC WP_00613379.1) and a nuclease (100% sequence identity to *E. coli* nuclease WP_00635769.1). Contig 5 carries the nickel ABC transporter protein NikA, common to the *S. sonnei* plasmids but has additional hypothetical proteins, and contig 6 consists of non-coding DNA common to the *S. sonnei* plasmids.

## Discussion

aEPEC are emerging enteropathogens that have been detected in both humans and animals globally. However, aEPEC are a heterogenous pathotype regarding their serotypes, STs, virulence factors and ability to cause disease. *E. coli* ST302 strains are aEPEC that have been isolated from both healthy and diseased animal and human hosts, yet no study to date has focused on this ST. Here we present the first closed chromosome and two circularized plasmids (pMLST types HI2:ST3 and FIB) from an ST302 strain; F2_18C, isolated from a healthy pig from Australia. Additionally, we compare F2_18C virulence and AMR gene cargo with other *E. coli* ST302 strains from a range of hosts including a human isolate, using Illumina short-read gene screening and compare F2_18C plasmids to those with high sequence identity.

Enteric colibacillosis is a significant cause of economic losses to pig industries worldwide (Fairbrother et al., 2005). The major causative pathotype is enterotoxigenic *E. coli* (ETEC), which is routinely screened for by veterinary diagnostic laboratories, and characterized by their fimbriae and enterotoxins (Fairbrother et al., 2005). However, though not routinely screened for, aEPEC (*eae*+, *stx*−, *bfp*−) are also known to cause enteric colibacillosis in swine (Fairbrother et al., 2005; Luppi, 2017) and García-Meniño et al. (2018) found aEPEC outnumbered ETEC as the causative agents in suckling piglets. Nevertheless, *eae*+ *E. coli* has been isolated from both healthy and diarrheic pigs (Krause et al., 2005; Fröhlicher et al., 2008; Malik et al., 2017), implying that a combination of additional VAGs, as well as host-related factors, and poor herd management and husbandry practices, are required to establish disease.

Despite being isolated from a healthy pig, aEPEC strain F2_18C possesses almost twice as many VAGs than non-pathogenic *E. coli* strain MG1655 (Figure 1). Many of these VAGs are carried on MGEs, including plasmids, prophage-like elements, and genomic islands. F2_18C carries an 82,910 bp plasmid, pF2_18C_FIB, with high sequence identity to FIB13 plasmids from EHEC ST17 strains (Figure 8). These plasmids carry VAGs implicated in EHEC virulence, including the pore-forming toxin *ehxA*, T2SS gene *etpD*, C1 esterase inhibitor cleaving protease *stcE*, and lipid A synthesis gene *msbB* (Somerville et al., 1999; Noll et al., 2018). Additionally, pF2_18C_FIB and one other ST17 plasmid carry the *katP* gene. *katP* encodes for a catalase-peroxidase thought to be expressed by pathogenic *E. coli* as a defensive mechanism against host-mounted oxidative stress (Uhlich, 2009). *katP* was also shown to be significantly associated with ST17 human isolates, rather than animal isolates (Söderlund et al., 2016), though in ST11s (O157:H7) *katP* has been found equally distributed among human and bovine isolates (González et al., 2017). Nevertheless, EHEC strains carrying both *katP and stcE* have been linked to high virulence (Kobayashi et al., 2013). pF2_18C_FIB and the ST17 FIB13 plasmids share homology with pO157 found in the most notorious of food-borne pathogens O157:H7 (Figure 8). However, they lack the adhesin *toxB* and the serine protease *espP*, which cleaves pepsin A and human coagulation factor V, hence thought to contribute to hemorrhagic colitis symptoms (Brunder et al., 1997).

In addition to pF2_18C_FIB, F2_18C has three contigs that did not circularize, but nonetheless have high sequence identity to *S. sonnei* ColRNAI plasmids. These plasmids carry colicin-10, and the colicin-10 immunity gene, which are also present in F2_18C contig 4 (Figure 9). Colicins are a type of bacteriocin produced by and active against *E. coli* (Fredericq, 1957). In the advent of plasmid-mediated colistin resistance, particularly in swine, colicins are currently being given consideration as viable alternatives to antibiotics (Ben Lagha et al., 2017). In particular, colicin E1 has been shown to inhibit the growth of swine ETEC strains *in vitro* (Gillor et al., 2004), and the addition of colicin E1 to pig feeds has been shown to decrease the severity and incidence of ETEC infections (Cutler et al., 2007). However, the colicin-10 immunity protein grants full protection against colicin E1 activity (Pilsl and Braun, 1995). Thus, a potential colistin-10 plasmid circulating amongst a pig population may dampen efforts in implementing colicin-E1 as an effective agent against ETEC and growing AMR in swine.

Like all aEPEC, F2_18C harbors a chromosomal LEE pathogenicity island that includes intimin (*eae*), an adhesin with a critical role in intestinal colonization. There are at least 30 distinct subtypes of intimin (Ramachandran et al., 2003; Xu et al., 2016), and it is thought that specific subtypes may be involved in tissue tropism and host specificity (Hartland et al., 1999; Torres et al., 2005). The ability of intimin to also bind β–integrins may be significant in this regard (Phillips and Frankel, 2000). The *eae* gene in F2_18C is subtype β1, which has been significantly correlated to disease in human patients (Xu et al., 2016), and is common in cattle and in healthy pigs (Ramachandran et al., 2003; Krause et al., 2005; Malik et al., 2006; Fröhlicher et al., 2008). In addition to LEE, F2_18C carries two other pathogenicity islands, ETT2, and PAI_*CL*__3_ (Figure 5). ETT2 shares homology to the *Salmonella* type 3 secretion system located on the *Salmonella* pathogenicity island 1 (SPI-1). Though it is thought unlikely that ETT2 functions as a secretion system, it is known to influence virulence by regulating VAGs expression (Zhou et al., 2014; Luzader et al., 2016), promoting motility, adhesion, biofilm formation and serum survival (Zhou et al., 2014; Shulman et al., 2018), and enhancing invasion and intracellular survival (Yao et al., 2009). While most *E. coli* strains possess ETT2 gene clusters, the vast majority have undergone extensive deletions and mutations (Ren et al., 2004; Zhou et al., 2014). However, intact ETT2s are prevalent in pathogenic porcine EHEC strains, particularly those associated with *stx_2__*e*_* (Cheng et al., 2012). F2_18C possesses the full complement of ETT2 genes (Figure 5A), however an IS1F insertion interrupts the *eivG* gene, and *eivC* gene is truncated. The latter observation may be particularly important as *eivC* is involved in motility, serum resistance and intra-macrophage survival, at least in avian pathogenic *E. coli* (APEC) (Wang et al., 2016).

Like ETT2, PAI_*CL*__3_ is rarely found in its entirety, and in most cases, all that remains are boundary genes (Girardeau et al., 2009). The complete PAI_*CL*__3_ locus is a hybrid genomic region of 35 ORFs, composed in part, of a *Yersinia pestis*-like hemolysin/adhesin genes cluster, OI-122 genes, including the VAG *pagC*, and OI-48 genes (Girardeau et al., 2009). PAI_*CL*__3_ was originally believed to be restricted to LEE-negative STEC only and a significant predictor of a virulent status (Girardeau et al., 2009). However, Bardiau et al. (2012) identified PAI_*CL*__3_ in 8 (11.3%) O26 EHEC and EPEC strains of human and bovine origins. These strains had the three left-side boundary OI-48 genes (Z1635-7), the hemolysin/adhesin cluster (S1–S4), and the three right boundary OI-48 genes (S15, Z1643-4), however most lacked S5–S14 genes (10 genes), including the five OI-122 genes. Conversely, the F2_18C PAI_*CL*__3_ isoform remains near intact, lacking only two right-side boundary genes (Figure 5B). To our knowledge, this is the first time PAI_*CL*__3_ has been described in swine and has potentially negative implications for its use as a marker for highly virulent strains.

The F2_18C chromosome is infected with 13 prophage elements (Figure 4), which carry a number of VAGs including non-LEE encoded effectors, iron acquisition genes and EHEC-associated gene Z2121 (Delannoy et al., 2013). The *aidA* gene was also found on a prophage element. This gene encodes for a membrane-bound autotransporter protein that functions as an adhesin that self-associates and also interacts with Ag43, another surface adhesin, to promote biofilm formation (Sherlock et al., 2004). An *aidA-*deletion mutant ETEC strain was shown to lose the ability to colonize and induce disease in swine emphasizing the important role it plays in pathogenesis (Fairbrother et al., 2005). Importantly, *aidA* was one a several genes that resulted in a false-negative identification by short-read gene screening (Table 1). Bacteriophages are the most abundant life-form on earth (Brüssow and Hendrix, 2002) and are widespread in sewage effluent. Given the propensity of aEPEC to be converted to EHEC by the introduction of Shiga toxin*-*carrying lambdoid bacteriophages (Eichhorn et al., 2018), F2_18C was checked for known *stx1* and *stx2*-phage integration sites and was found to have several vacant. It is theorized that aEPEC are the progenitors of EHEC (Kyle et al., 2012; Eichhorn et al., 2018), and so it is therefore prudent to monitor their VAG cargo, particularly EHEC-associated VAGs, and factor in aEPEC detection into EHEC control measures. That being said, though the virulence potential of F2_18C appears high, no STEC ST302 strains have been observed, and F2_18C was isolated from a healthy pig, therefore it was also prudent to take note of EPEC/EHEC VAGs missing in this strain (Table 2). Nonetheless, serotype O108:H9 *E. coli* such as F2_18C are frequently isolated from pigs with gastrointestinal disease (Malik et al., 2006; Kleta et al., 2014) and from healthy swine (Krause et al., 2005; Fröhlicher et al., 2008) suggesting that they may be opportunistic pathogens that cause diarrheal disease in swine during stressful episodes such as weaning.

When compared to other *E. coli* ST302 strains, F2_18C had more VAGs than most, with the notable exception of two O182:H9 strains 29563 and 29396. These two strains were highly homologous in their VAG profile (Figure 3) and were the only two in addition to F2_18C to carry the EHEC plasmid marker genes *ehxA, etpD*, and *katP.* However, unlike F2_18C, 29563 and 29396 appear to have acquired several extraintestinal *E. coli* (ExPEC) VAGs, including *cvaA, iha, shiD* and genes encoding aerobactin iron acquisition. Additionally, 29563 and 29396 uniquely carry *nleB1, nleE* and *espL2/ent*, which together with *eae*, represent a strong signature for human pathogenic EHEC (Bugarel et al., 2011). Incidentally, both maximum-likelihood and SNP-based phylogenetic analyses (Figures 2, 3), place F2_18C, 29563 and 29396 in a distinct off-shoot from other *E. coli* ST302 strains and may represent an evolutionary pathway shaped by VAG acquisition. When compared to 76 *E. coli* genomes of varying STs, serotypes, phylogroups and hosts, *E. coli* ST302 strains were most closely related to EHEC O145:H28 strains, two of which, RM12581 and RM12761, were associated with major food-borne outbreaks (Cooper et al., 2014) (Figure 2).

As well as being an ST302 with one of the highest virulence potentials, F2_18C also carries the most AMR genes acquired through HGT (Figure 3). This is, in all likelihood, due to F2_18C being only one of two ST302 strains to carry an intact class 1 integron marker gene *intI1.* Integrons are MGEs capable of capturing and mobilizing functional gene cassettes, and class 1 integrons have played a pivotal role in disseminating antibiotic resistance worldwide (Hall and Collis, 1998). F2_18C carries ARGs conferring resistance to aminoglycoside (*aadA1, aadA2*), chloramphenicol (*cmlA*), kanamycin and neomycin [*aph(3′)-IIa*], streptomycin (*strA, strB*), sulphonoamide (*sul1, sul3*), tetracycline (*tetA, tetR*) and trimethoprim (*drfA12*). Strain 267792, the only other ST302 to carry an intact *intI1* gene, was isolated from a human in the United Kingdom, and possesses a different AMR profile to F2_18C, carrying additional genes conferring resistance to macrolides (*mphA*) and quinolone (*qnrB19*), but lacking resistance genes to chloramphenicol, streptomycin, kanamycin and neomycin. Limitations of our ST302 VAG and ARG comparisons include the reliance of short-read data, which we previously mentioned can provide false-negatives, the limited number of ST302 genomes currently available and the limited metadata provided for strains, including the host, isolation date and location for some strains, and host disease status for all strains, except F2_18C. We were able to map all F2_18C horizontally transferred ARGs to a 274, 059 bp circular plasmid, pF2_18C_HI2 (Figure 6A). This plasmid was typed by pMLST as HI2:ST3, a lineage known as a major contributor in transferring complex class 1 integrons conferring AMR (Zhao H. et al., 2018) pF2_18C_HI2 is very similar to three HI2:ST3 plasmids circulating in Australian pig farms (Dyall-Smith et al., 2017; Billman-Jacobe et al., 2018; Wyrsch et al., 2019) (Figure 6A). pF2_18C_H12 carries the same AMR profile as pSDE-SvHI2, isolated from a severe ETEC strain (Wyrsch et al., 2019), and differs to a pSDC-F2_12BHI2, isolated from a commensal *E. coli* strain (Wyrsch et al., 2019), and pSTM6-275, isolated from *Salmonella enterica* subsp. *Enterica* Typhimurium (Dyall-Smith et al., 2017), by lacking aminoglycoside resistance gene *aph(3′)-Ia* and fluoroquinolone resistance genes *oqxAB* in the former, and beta-lactam resistance gene *blaTEM-1B* in the latter.

We compared the AMR cargo of 34 HI2:ST3 plasmids (Figure 7), five from Australia, one from an unspecified origin, and 28 from Asia, the vast majority of which originated in China. Perhaps due to restrictions on live animal imports and stringent antibiotic usage practices, HI2:ST3 originating from Australia do not, yet, carry any ARG conferring resistance to extended spectrum beta-lactams (ESBLs), colistin, fosfomycin or rifampicin. This is in stark contrast to HI2:ST3 plasmids originating from Asia, in which ESBL genes, such as *bla_*CTX–M–*__14__,–__55__,–__65__,–__199_, bla_*OXA–*__1__, –__10_*, and fosfomycin gene *fosA3*, are prevalent. Moreover, 54% of HI2:ST3s from China carry colistin-resistance gene *mcr-1*. The increased global usage of colistin in agriculture coincided with the rising prevalence of multidrug resistant Enterobacteriaceae (Falagas and Michalopoulos, 2006). China, being the world’s largest producer of pork, is incidentally also the world’s largest user and producer of colistin, making up to 73% of global colistin production and driving global demands for agricultural colistin usage to an anticipated 11,942 tons per annum in 2015 (Liu et al., 2016). Resistance to colistin was originally believed to only occur from relatively rare mutations in a lipid A modification gene (Falagas et al., 2010). Although, the first plasmid mediated-colistin resistance was identified in swine from China in 2015 (Liu et al., 2016), it has now been identified in over 31 countries across five continents from a range of animal and human hosts, sparking worldwide concern, as colistin is used clinically as a last resort for multidrug resistant Gram-negative infections (Holmes et al., 2016). The colistin resistance gene *mcr-1* is mobilized by the transposon Tn*6330* (IS*Apl*1-*mcr-1*-orf-IS*Apl*1) which can generate a circular intermediate (Is*Apl*1-*mcr-1*-orf) (Li et al., 2017; Wang et al., 2018). This circular intermediate is capable of inserting itself into the highly conserved HI2:ST3 backbone (Figure 6B). China banned the use of colistin in pig feeds in 2016 (Walsh and Wu, 2016), however, due to the sheer number of ARG carried by these plasmids, as well as large metal-resistance regions (Figure 6), researchers fear that selective-pressures, through the use of other antibiotics and metal feed additives, will still favor these plasmids, and colistin resistance will continue to spread.

## Conclusion

Despite the limited number of publicly available genome sequences it is evident that ST302 *E. coli* should be broadly considered aEPEC strains that may reside in healthy hosts or cause opportunistic disease. Of concern is the apparent divergence of strains encoding additional virulence traits including those typical of EHEC and ExPEC, which may yield more severe disease presentation. It would be of great benefit to phenotypically characterize these strains in order to fully understand their potential for pathogenicity in humans and animals. Furthermore, the acquisition of MDR plasmids in lineages with pathogenic potential is a major issue for global public health as antimicrobial use may co-select for virulence traits. Our study highlights *E. coli* ST302 as a potentially emerging pathogen that should be monitored in the context of public health. However, further genomic epidemiology studies of *E. coli* ST302 within a One Health framework are required to fully understand this lineage, its virulence potential, and inform genomic surveillance in the future.

## Data Availability Statement

The datasets generated for this study can be found in the GenBank repository under the assession numbers CP043542, CP043545, CP043544, and CP043543.

## Author Contributions

VJ performed the genomic analyses, created the figures, and drafted the manuscript. CR performed the genome assemblies and SNP phylogeny, and assisted in the analyses. TC provided the strain F2_18C and performed the antibiotic sensitivity testing. SD initiated, funded, and supervised the study. All authors contributed to the manuscript revisions and gave final approval for publication.

## Conflict of Interest

The authors declare that the research was conducted in the absence of any commercial or financial relationships that could be construed as a potential conflict of interest.

## Footnotes

1 https://www.bioinformatics.babraham.ac.uk/projects/fastqc/

2 http://www-is.biotoul.fr

3 http://www.pathogenomics.sfu.ca/islandviewer/

4 https://www.patricbrc.org/

5 http://phaster.ca/

6 http://tree.bio.ed.ac.uk/software/figtree/

7 https://itol.embl.de/

8 https://github.com/tseemann/snippy

9 https://github.com/sanger-pathogens/gubbins

10 https://github.com/sanger-pathogens/snp-sites

11 https://cge.cbs.dtu.dk/services/MLST/

12 https://cge.cbs.dtu.dk/services/SerotypeFinder/

13 http://enterobase.warwick.ac.uk/

14 http://enterobase.warwick.ac.uk/

15 https://cge.cbs.dtu.dk/services/ResFinder/

16 https://card.mcmaster.ca/home

17 https://cge.cbs.dtu.dk/services/VirulenceFinder/

18 http://www.mgc.ac.cn/VFs/

19 https://cge.cbs.dtu.dk/services/PlasmidFinder/

20 https://ccb-microbe.cs.uni-saarland.de/plsdb/

21 http://brig.sourceforge.net/

22 http://darlinglab.org/mauve/mauve.html

## Supplementary Material

DATA S1 *E. coli* strain F2_18C genome annotations.

DATA S2 ST302 SNP analysis.

DATA S3 *E. coli* straim F2_18C PHASTER analysis.

TABLE S1 *E. coli* strain F2_18C gene functional assignments and specialty genes provided by PATRIC.

TABLE S2 *E. coli* strains used in ST302 phylogenetic analysis.

TABLE S3 ST302 pangenome analysis.

TABLE S4 ST302 short-read gene screening output by Ariba.

TABLE S5 IslandViewer output for *E. coli* strain F2_18C.
